# Renal Protective Effect of Xiao-Chai-Hu-Tang on Diabetic Nephropathy of Type 1-Diabetic Mice

**DOI:** 10.1155/2012/984024

**Published:** 2012-03-07

**Authors:** Chun-Ching Lin, Liang-Tzung Lin, Ming-Hong Yen, Juei-Tang Cheng, Chung-Hsi Hsing, Ching-Hua Yeh

**Affiliations:** ^1^School of Pharmacy, College of Pharmacy, Kaohsiung Medical University, Kaohsiung 807, Taiwan; ^2^Graduate Institute of Natural Products, College of Pharmacy, Kaohsiung Medical University, Kaohsiung 807, Taiwan; ^3^Department of Pediatrics, IWK Health Centre, Halifax, NS, Canada B3K 6R8; ^4^Institute of Medical Science, College of Health Science, Chang Jung Christian University, Tainan 71101, Taiwan; ^5^Department of Anesthesiology, Chi Mei Medical Center, Tainan 710, Taiwan; ^6^Department of Anesthesiology, College of Medicine, Taipei Medical University, Taipei 110, Taiwan

## Abstract

Xiao-Chai-Hu-Tang (XCHT), a traditional Chinese medicine formula consisting of seven medicinal plants, is used in the treatment of various diseases. We show here that XCHT could protect type-1 diabetic mice against diabetic nephropathy, using streptozotocin (STZ)-induced diabetic mice and high-glucose (HG)-exposed rat mesangial cell (RMC) as models. Following 4 weeks of oral administration with XCHT, renal functions and renal hypertrophy significantly improved in the STZ-diabetic mice, while serum glucose was only moderately reduced compared to vehicle treatment. Treatment with XCHT in the STZ-diabetic mice and HG-exposed RMC resulted in a decrease in expression levels of TGF-**β**1, fibronectin, and collagen IV, with concomitant increase in BMP-7 expression. Data from DPPH assay, DHE stain, and CM-H_2_DCFDA analysis indicated that XCHT could scavenge free radicals and inhibit high-glucose-induced ROS in RMCs. Taken together, these results suggest that treatment with XCHT can improve renal functions in STZ-diabetic mice, an effect that is potentially mediated through decreasing oxidative stress and production of TGF-**β**1, fibronectin, and collagen IV in the kidney during development of diabetic nephropathy. XCHT, therefore merits further investigation for application to improve renal functions in diabetic disorders.

## 1. Introduction

Increasing prevalence of nephropathy and/or end-stage renal disease (ESRD) in diabetic patients is becoming a serious social and health problem worldwide [1, 2]. Hyperglycemia is considered as the main factor to induce diabetic nephropathy (DN), and clinical strategies for management of DN include glycemic control and blood pressure regulation [3, 4]. However, current therapeutic effect for DN remains unsatisfactory, thus resulting a year-by-year increase in the number of diabetic patients with nephropathy [5]. Pathological changes of DN are characterized by structural abnormalities including renal-cell hypertrophy, increase in thickness of glomerular basement membranes, and progressive accumulation of extracellular matrix components [6]. 

Due to hyperglycemia, diabetes exhibits high oxidative stress which depletes the activity of antioxidative defense system and in turn promotes* de novo* free radicals generation; thus, strategies to reduce oxidative stress in diabetes mellitus may exert favorable effects on the progression of diabetic glomerulosclerosis [7, 8]. Hyperglycemia and oxidative stress during DN induce abnormal production and stimulation of TGF-*β*1-resident renal cells [4, 6, 8, 9]. TGF-*β*1 causes augmented deposition of extracellular matrix proteins, such as collagen IV and fibronectin, in the glomeruli, thus inducing mesangial expansion and glomerular basement membrane thickening [1]. The renal protective protein, bone morphogenetic protein-7 (BMP-7), can reduce glomerular and tubulointerstitial fibrosis and prevent the pathogenesis in diabetic nephropathy [10, 11]. Expression or treatment with recombinant BMP-7 has been shown to improve renal damage from hyperglycemia-induced oxidative stress in the kidney of diabetic animals [10, 12].

Xiao-Chai-Hu-Tang (Japanese name, Shosaiko-to) is a herbal drug formula widely used in traditional Chinese medicine and Japanese *kampo* medicine. XCHT consists of a mixture of seven different medicinal plants (Table 1) and has several experimentally proven pharmacological activities, including prevention of experimental hepatotoxicity [13], immunomodulatory effect [14–16], antineoplastic activity [17] and promotion of liver regeneration [18]. XCHT has also been documented to decrease fibrogenic protein expression, including TGF-*β*1 and collagen, to inhibit hepatic fibrosis [19–21]. However, it remains unclear whether XCHT has any renal protective activity during diabetic nephropathy and could prevent streptozotocin (STZ)-induced type-1 diabetic mice from renal fibrosis. We, therefore, investigated the treatment effect of XCHT on STZ-induced type-1 diabetic mice and high-glucose (HG)-exposed rat mesangial cell (RMC) models. Expression levels of TGF-*β*1, fibronectin, collagen IV, and BMP-7 as well as antioxidative effects of XCHT treatment were examined in order to explore the renal protective effect and mechanism of XCHT in diabetic nephropathy.

## 2. Materials and Methods

### 2.1. Preparation of Xiao-Chai-Hu-Tang Extract

The seven different constituent medicinal plants of Xiao-Chai-Hu-Tang (XCHT) are listed in Table 1. They were purchased from a herbal grocery in Kaohsiung city, Taiwan. The authenticity of these ingredients was confirmed by Dr. M. H. Yen (Graduate Institute of Natural Products, Kaohsiung Medical University, Taiwan) using histological technique. XCHT was prepared by mixing the appropriate proportions of the dried constituents (Table 1). To prepare the aqueous extract of XCHT, 108 g of the dried material was extracted with 1 liter of boiling water for 2 h. After the supernatant of the first extraction was removed, an additional 1 liter of distilled water was added and the sample was further subjected to another 2 h of extraction. The same procedure was repeated for 3 times. The decoction obtained from the three extractions was combined, filtered, concentrated, and lyophilized. The yield of the dried extract was about 38%, which was collected and stored at −20°C until use.

### 2.2. Animal Model for Diabetic Nephropathy

BALB/c mice, 8-week old, 18–20 g in weight, were obtained from the Animal Center of National Cheng Kung University Medical College. STZ-diabetic mice were induced by intraperitoneal (i.p.) injecting STZ (200 mg/kg) (Sigma-Aldrich Inc., Saint Louis, MO, USA) into fasting mice as described previously [22]. Mice were considered to be diabetic if they had elevated plasma glucose concentrations of 350 mg/dL. All experiments were carried out at 9 weeks after STZ injection. STZ-diabetic mice were considered to have diabetic nephropathy (DN) when they had elevated levels of urea nitrogen (BUN) and creatinine. All animal procedures were performed according to the *Guide for the Care and Use of Laboratory Animals of the National Institutes of Health*. Chang Jung Christian University approved the animal care protocol (IACUC approval no. CJCU-99-002) for the experiments performed in this study. STZ-diabetic mice were treated with 200 mg/kg of XCHT twice a day for four weeks. The vehicle (distilled water) used to disperse XCHT was given at the same volume. The STZ-diabetic mice were divided into 3 groups (*n* = 6), respectively: (1) Vehicle-treated normal mice; (2) Vehicle-treated STZ-diabetic mice and (3) XCHT-treated STZ-diabetic mice. At the end of the 4-week treatment, mice were euthanized by cervical dislocation after pentobarbital (40 mg/kg, i.p.) anesthesia, and blood was collected from their abdominal aorta for renal function analysis. Kidneys were dissected and rinsed with cold isotonic saline and then weighed, frozen in liquid nitrogen, and then kept for storage at −80°C for further analysis. An index of renal hypertrophy was estimated by comparing the weight of the left kidney to the body weight.

### 2.3. Blood Glucose and Renal Functions Determination

Blood samples were collected before the treatments and one hour after the last treatment for estimating the levels of plasma glucose, BUN, and creatinine. Blood samples from the mice were centrifuged at 3,000 g for 10 min. Samples were incubated with reagents from glucose, BUN, or creatinine kits (AppliedBio assay kits; Hercules, CA, USA), and the levels of blood glucose, BUN, and serum creatinine were then assessed by an autoanalyzer (Quik-Lab, Ames, Miles Inc., Elkhart, Indiana, USA), with samples run in duplicate.

### 2.4. Renal Histological Analysis

For morphometric analysis, the kidney was removed and embedded in paraffin to prepare 4-*μ*m tissue slices. The tissue slices were stained with periodic acid-Schiff (PAS). The mesangial expansion index (MEI) was scored in four levels from 0 to 3, with the index scores defined as follows [23]: 0, normal glomeruli; 1, matrix expansion occurred in up to 50% of a glomerulus; 2, matrix expansion occurred in 50 to 75% of a glomerulus; 3, matrix expansion occurred in 75 to 100% of a glomerulus. Scores were assigned for at least 30 glomeruli from kidney slices from each mouse, and the means were calculated. Each slide was scored by a pathologist who was unaware of the experimental details.

## 3. Immunohistochemical Staining

Kidney sections were dewaxed and hydrated through graded ethanol solutions and then rinsed with PBS. After treatment for 5 min in 3% H_2_O_2_ and 30 min in blocking agent, slides were incubated with primary antibodies of TGF-*β*1, fibronectin, collagen IV, or BMP-7 (Santa Cruz, USA) diluted to 1 : 50 in PBS for 16 h at 4°C. Then, slides were washed three times with PBS and incubated for 30 min with biotin-conjugated secondary antibodies (Vector, UK) for 1 h. The slides were washed again with PBS and the signal was amplified with Vectastain ABC kit (Vector, UK). Finally, the samples were developed using diaminobenzidine (DAB). Hematoxylin was applied as the counter stain. 

### 3.1. RMCs Cell Culture

Rat mesangial cell (RMC) line was purchased from the American Type Culture Collection (Manassas, VA, USA). RMCs were cultured in Dulbecco's modified Eagle's medium (DMEM; Gibco-BRL, Gaithersburg, MD) containing 5 mmol/L glucose supplemented with 15% FBS and antibiotics, and maintained at 37°C in 95% air, 5% CO_2_. We added 25 mmol/L glucose (final concentration 30 mmol/L) into serum-free DMEM for high-glucose (HG) medium. DMEM without FBS and additional glucose concentration solution were used as control medium. When the cell monolayer reached about 60% confluency, culture medium was replaced with control or HG medium with different treatments and then cultured for 24 h. XCHT (1,5, 10, 50 *μ*g/mL) and the free radical scavenger tiron (10 mmol/L; 4,5-dihydroxyl-1,3-benzene disulfonic acid; Sigma-Aldrich Inc., Saint Louis, MO, USA) [10, 12] were used for the treatments.

### 3.2. Reverse Transcriptase Polymerase Chain Reaction (RT-PCR)

RT-PCR was performed to determine the mRNA expression levels of TGF-*β*1, fibronectin, collagen IV, and BMP-7. Total RNA of mouse kidney, or RMCs was extracted using Trizol solution (Life Technologies) and subjected to reverse transcription with StrataScript H-reverse transcriptase (Stratagene, La Jolla, CA) to generate cDNA. Gene-specific primer pairs (sense and antisense, resp.) used are as follows: TGF-*β*1,  F5′-CGGCAGCTGTACATTGACTT  and R5′-TCAGCTGCACTTGCAGGAG;  fibronectin, F5′-GAATCCAGTCCACAGCCATTCCT and R5′- CCGCAGGTTGGA  TGGTGCGTG;  collagen  IV, F5′- CTGGCACAAAAGGGACGAG and R5′-ACGTGGCCGAGAATTTCACC;  BMP-7,  F5′-  CTGGATGGGCAGAGCATCAA-3′ and R5′- TGGTTGGTGGCGTTC  ATGTA-3′;  *β*-actin, F5′-GCTGGAAGGTGGACAGCGAG-3′ and  R5′-TGGCATCGTGATGGACTCCG-3′. PCR products were electrophoresed on 1.5% agarose gels and stained with ethidium bromide. *β*-actin amplification was used as an internal control.

### 3.3. Western Blot Analysis for Kidney and RMCs

TGF-*β*1, fibronectin, collagen IV, and BMP-7 expression levels in mouse kidney or RMCs were determined by Western blotting analysis. RIPA buffer was used to extract total protein. For Western analysis, proteins were separated by SDS-PAGE, transferred, and immobilized on a nitrocellulose membrane. The membrane was blocked with 5% nonfat dry milk in phosphate-buffered saline containing 0.1% Tween 20 (PBS-T) for 2 h at room temperature. The membrane was then washed in PBS-T and hybridized with primary antibodies diluted to proper concentration in PBS-T for 16 h. Specific antibodies (Santa Cruz Biotechnology Inc., Santa Cruz, CA, USA) for TGF-*β*1, fibronectin, collagen IV, BMP-7 (1 : 200 dilution), and *β*-actin (1 : 1000 dilution) were used. Incubation with secondary antibodies and detection of the antigen-antibody complex were performed using the ECL kit (Amersham Biosciences, UK). 

### 3.4. DPPH Radical Scavenging Assay

The antioxidant activity of XCHT and ascorbic acid (vitamin C) was measured in terms of 1,1-diphenyl-2-picrylhydrazyl (DPPH) (Sigma-Aldrich Inc., Saint Louis, MO, USA) free radical scavenging ability. Vitamin C, an antioxidant, was used as a positive control. The highest tested concentration of Vitamin C was considered as 100% of scavenging activity. A solution of XCHT at different concentrations was placed in a cuvette and 1 mL of DPPH radical in methanol solution (23.7 *μ*g/mL) was added, followed by incubation for 30 min. The decrease in absorbance at 517 nm was determined with a spectrophotometer. All determinations were performed in triplicates. The percent inhibition of DPPH radical by the samples was calculated according to the following formula:

(1) $$\%\;\text{of}\;\text{scavenging}\;\text{of}\;\text{DPPH}\;=\left[1-\frac{A_{\left(s\right)}}{A_{\left(c\right)}}\;\right]\times \;100,$$

where *A*_(*c*)_ is the absorbance of the control (without sample), and *A*_(*s*)_ is the absorbance of the sample at  *t* = 30 min. 

### 3.5. Intracellular ROS Detection

#### 3.5.1. DHE Staining

RMCs (10,000 cells) were seeded on 12-well cell culture plates and after 24 h growth, the culture medium was replaced with control (normal glucose, NG) or high glucose (HG) medium in the presence or absence of tiron (antioxidant control; 10 mmol/L) or XCHT (20 *μ*g/mL) for another 24-h incubation before being harvested. The RMCs were fixed with 3.7% paraformaldehyde for 30 min, washed three times with PBS, then stained with 5 *μ*mol/L of dihydroethidium (DHE; Invitrogen) which acts as a fluorescent indicator of ROS generated in response to the described treatment. Images were acquired with an Olympus IX70 fluorescence microscope fitted with an Olympus America camera, and analyzed with MagnaFire 2.1 software.

#### 3.5.2. CM-H_2_DCFDA Analysis

Intracellular oxidative stress was also measured by dichlorodihydrofluorescein diacetate oxidation. Briefly, 5,000 RMCs were plated in 96-well plates overnight and washed twice with HBSS before experiments. The cells were exposed to 20 *μ*M 5-(and-6)-chloromethyl-2′, 7′-dichlorodihydrofluorescein diacetate, acetyl ester (CM-H_2_DCFDA) (Invitrogen Life Technologies, Carlsbad, CA, USA) for 1 h and then treated with normal-glucose (NG) or HG medium combining with vehicle or different concentrations of XCHT or 10 *μ*mol/L of tiron. The fluorescence was read immediately at wavelengths of 485 nm for excitation and 530 nm for emission on a fluorescence plate reader (Fluoroskan Ascent; Thermo Electron Corporation, Milford, MA, USA). The levels of ROS were calculated as percent increases compared with the control, and the control was normalized to 100% of the basal level.

### 3.6. Statistical Analysis

Results were expressed as mean ±   SD. Statistical analysis was carried out by using ANOVA analysis and Dunnett post hoc analysis. Statistical significance was achieved if *P* < 0.05.

## 4. Results

### 4.1. XCHT Oral Administration Improves Renal Functions in STZ-Diabetic Mice

**Figure 1:**
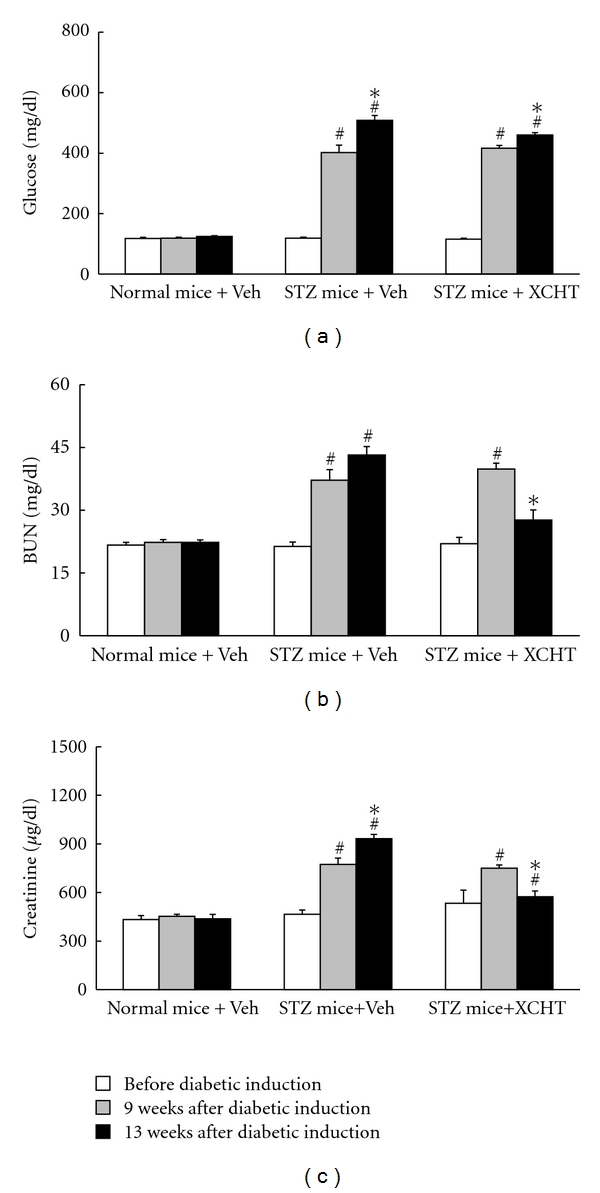
Effects of XCHT on serum levels of glucose (a), BUN (b), and creatinine (c) in normal or STZ-diabetic mice after a 4-week XCHT (200 mg/kg) or vehicle (Veh) treatment. Mice were first induced with STZ for 9 weeks to induce DN and then orally administered with treatments. Values (mean ± SD) were obtained from 6 mice for each test group. ^#^ *P* < 0.05 compared to the value of vehicle-treated normal mice. *P < 0.05 values from after 4 weeks of treatment compared to values prior treatment.

Mice were injected with STZ to induce type-1-like diabetic disease [22, 24]. After 9 weeks, the STZ-injected mice developed typical features of diabetes nephropathy, with serum levels of glucose, BUN, and creatinine being significantly higher than those observed in normal mice (*P* < 0.05) (Figures 1(a), 1(b) and 1(c)) [25, 26]. In the absence of intervention (vehicle only), these three blood chemistry parameters in the vehicle-treated STZ-diabetic mice increased significantly when comparing with levels prior to treatment initiation. In contrast, STZ-diabetic mice that received XCHT had modest reduction in serum glucose but a significant decrease in serum levels of BUN and creatinine compared to that in vehicle control treatment after the 4-week oral administration (*P* < 0.05) (Figures 1(a), 1(b) and 1(c)).

**Figure 2:**
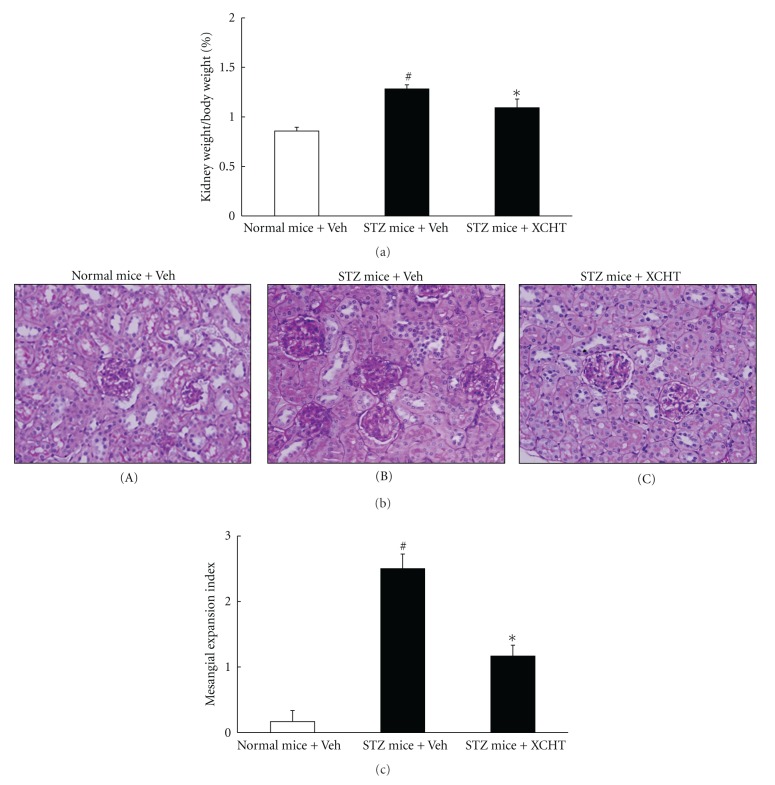
Effects of XCHT on kidney hypertrophy of normal or STZ-diabetic mice after a 4-week XCHT (200 mg/kg) or vehicle (Veh) treatment. (a) The kidney/body weight ratio. Values (mean ± SD) were collected from 6 mice for each test group. (b) Representative photomicrographs (original magnification, 200x) of PAS-stained kidney sections from STZ-diabetic mice receiving 4 weeks of XCHT or vehicle treatment; (A) normal mice treated with vehicle, (B) STZ-diabetic mice treated with vehicle, and (C) STZ-diabetic mice treated with XCHT. (c) Expansion of the glomerular matrix was scored as described in the text, and an average value was obtained from analyses of 30 glomeruli in each mouse kidney. ^#^ *P* < 0.05  values of STZ-diabetic mice compared to that of the normal mice. *P < 0.05 values of XCHT-treated STZ-diabetic mice compared to values from vehicle-treated STZ-diabetic mice.

At the end of the *in vivo* experiment, vehicle-treated STZ-diabetic had enlarged kidneys, with the ratio of kidney weight to body weight being significantly higher than that of normal mice (Figure 2(a)). XCHT treatment, however, reduced the degree of renal hypertrophy in the STZ-diabetic mice (*P* < 0.05). When performing histological analysis in the vehicle-treated normal mice, the PAS stain shows nearly normal glomerular structure with only mild mesangial expansion and no significant tubular damage (Figure 2(b) A). In contrast, there is an increase in mesangial matrix, mesangial cellularity, and capillary basement membrane thickening in the diabetic mice that received vehicle treatment (Figure 2(b) B). In particular, remarkable changes were observed in the tubulointerstitial areas, including tubules dilation and lined by flattened epithelium. The glomerular hypertrophy is also prominent in the vehicle-treated STZ-diabetic mice (Figure 2(b) B). These histological changes, however, improved in the diabetic mice administered with XCHT, which showed reduction in mesangial expansion, basement membrane thickening, glomerular hypertrophy, and tubular damage (Figure 2(b) C). These observations are also reflected in the index of the glomerular matrix expansion assessed in each mice group (Figure 2(c)). Thus, XCHT treatment can effectively improve renal functions and prevent kidney injury in diabetic mice induced by STZ.

## 5. XCHT  Treatment  Reduces  TGF-***β***1,  Fibronectin,  and  Collagen  IV Expression  While  Increasing  BMP-7  Levels  in  STZ-Diabetic  Mice

**Figure 3:**
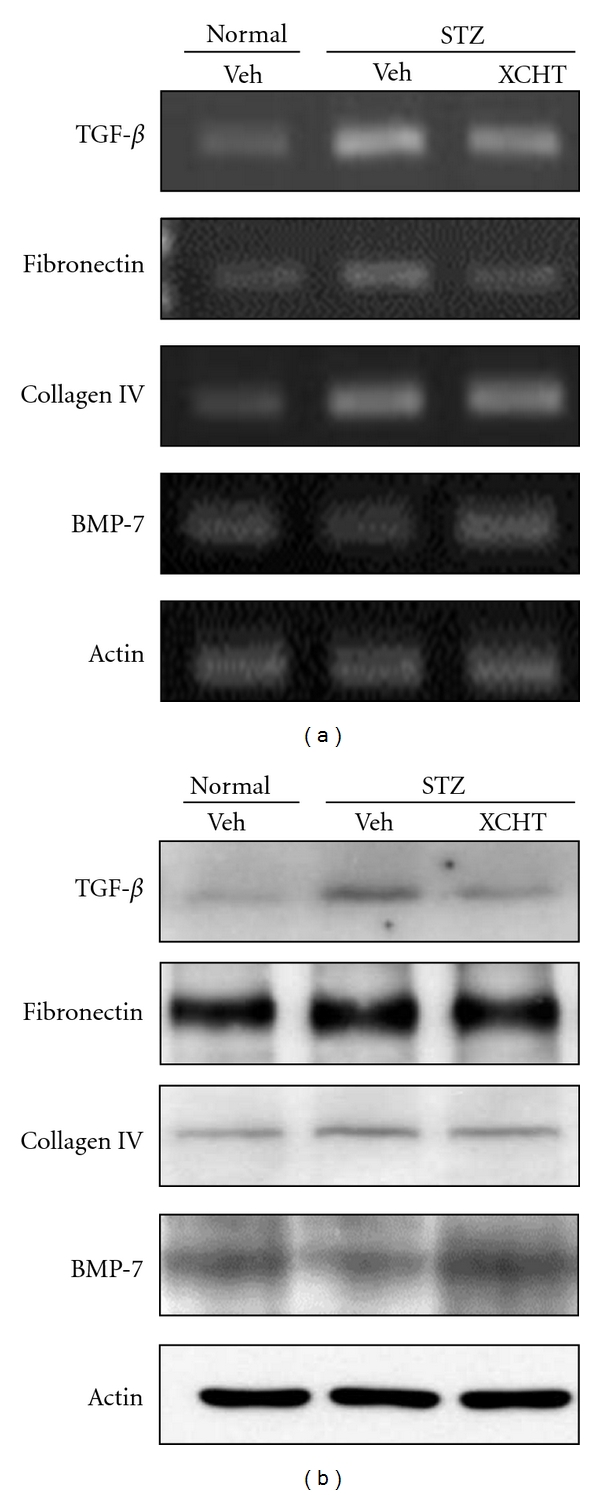
Effect of XCHT on TGF-*β*1, fibronectin, collagen IV, and BMP-7 expression in the kidney of normal or STZ-diabetic mice after 4 weeks of treatment (*n* = 6 in each group). (a) The mRNA expression of TGF-*β*1, fibronectin, collagen IV, and BMP-7 in the kidney of mice was detected using RT-PCR. *β*-actin mRNA expression was included as internal control. (b) Western blot analysis of TGF-*β*1, fibronectin, collagen IV, and BMP-7 expression in the kidney of the test mice. Representative data are shown.

In order to explore the underlying mechanisms in XCHT's renal protective effects against diabetic nephropathy, changes in the mice kidney expression of TGF-*β*1, fibronectin, collagen IV, and BMP-7 were assessed using RT-PCR and Western blot analyses. Vehicle-treated STZ-diabetic mice showed higher expression of TGF-*β*1, fibronectin, and collagen IV than in vehicle-treated normal mice (Figures 3(a) and 3(b)). Furthermore, due to the diabetic induction, the expression of BMP-7 in the diabetic mice receiving control treatment was lower than that in the vehicle-treated normal mice. In contrast, after 4 weeks of XCHT treatment in STZ-diabetic mice, kidney expression of these markers reverted to levels similar to vehicle-treated normal mice, with augmented BMP-7 and reduced levels of TGF-*β*1, fibronectin, and collagen IV expression (Figures 3(a) and 3(b)).

**Figure 4:**
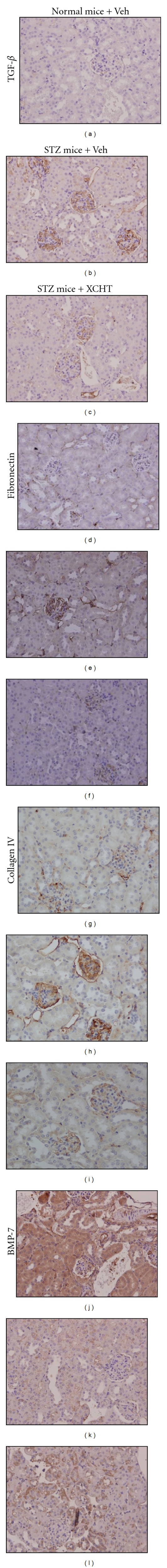
Renal immunostaining for TGF-*β*1 (a)–(c), fibronectin (d)–(f), collagen IV (g)–(i), and BMP-7 (j)–(l) expression in normal or STZ-diabetic mice receiving 4 weeks of XCHT treatment (*n* = 6 per group). Original magnification, 200x. Representative micrographs are shown.

To further validate the above results, renal immunostaining for TGF-*β*1, fibronectin, collagen IV, and BMP-7 expression was carried out on kidneys of the test mice. As shown in Figure 4, the STZ-diabetic mice exhibited greater TGF-*β*1 immunostaining in the glomeruli (Figure 4(b)) whereas XCHT-treated mice showed reduced TGF-*β*1 protein (Figure 4(c)). The expression of fibronectin and collagen IV was observed to accumulate in the glomeruli and interstitial space of STZ-diabetic mice (Figures 4(e) and 4(h)) relative to that of the normal mice (Figures 4(d) and 4(g)). Following the 4-week treatment, however, the extent of fibronectin and collagen IV expression in the glomeruli and interstitial space of XCHT-treated STZ-diabetic mice (Figures 4(f) and 4(i)) was clearly less than in the vehicle-treated STZ-diabetic mice (Figures 4(e) and 4(h)). Compared to the strong immunostaining of BMP-7 expression in normal mice (Figure 4(j)), STZ-diabetic mice showed reduced immunostaining of BMP-7 (Figure 4(k)) and 4-week treatment with XCHT could reverse the pathophysiological effect by enhancing its expression (Figure 4(l)). From these results, it appears that XCHT mediates its renal protective effect on the pathophysiology of diabetes in part by reducing profibrotic protein expression and increasing BMP-7 level.

### 5.1. XCHT Inhibits Oxidative Stress Induced by High-Glucose Exposure in RMCs and Reduces Profibrotic Factors While Increasing BMP-7 Expression

**Figure 5:**
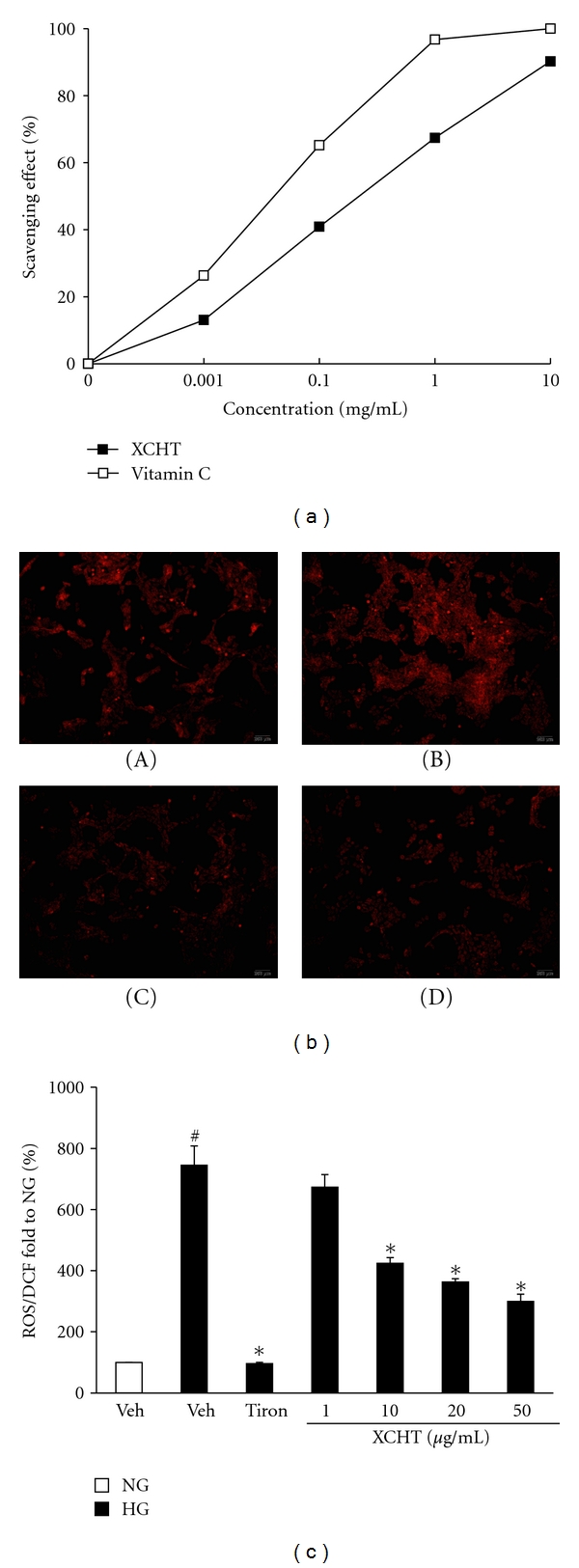
Effect of XCHT on free radicals in glucose-exposed RMCs. (a) Direct free radical scavenging activity of XCHT determined by the DPPH radical scavenging assay. Values represent the data of three independent experiments (SE < 0.05). Vitamin C was used as a positive control compound. (b) Intracellular visualization of ROS in RMCs under different treatments using DHE stains; (A) normal-glucose medium, (B) high-glucose medium, (C) high-glucose medium containing XCHT (20 *μ*g/mL), (D) high-glucose medium containing tiron (antioxidant control; 10 mmol/L). (c) Quantitation of ROS generation in RMCs using CM-H_2_DCFDA assay. RMCs were cultured in normal glucose (NG; 5 mmol/L) or high-glucose (HG; 30 mmol/L) medium in the presence of XCHT (1, 10, 20, and 50 *μ*g/mL) or tiron (10 mmol/L) for 24 h. ^#^ *P* < 0.05 compared to NG., *P < 0.05 compared to HG.

We have previously shown that exposing RMCs to high-glucose (HG) medium raises ROS production in these cells [12], and ROS induced by hyperglycemia can stimulate TGF-*β*1 and contribute to diabetes nephropathy [6]. To further determine whether oxidative stress is involved in XCHT's renal protective mechanism under diabetic condition, we evaluated the direct ROS scavenging effect of XCHT in HG-exposed RMCs. As shown in the DPPH radical scavenging assay (Figure 5(a)), XCHT could scavenge the free radicals directly in a concentration-dependent manner similar to the antioxidant vitamin C. The anti-oxidative ability of XCHT was further visualized by DHE stain (Figure 5(b)) and measured by CM-H_2_DCFDA staining (Figure 5(c)). In both instances, ROS was noted to increase in RMCs under high-glucose exposure compared with normal-glucose (NG) control (Figures 5(b) A, 5(b) B and 5(c)), but its levels were markedly inhibited by XCHT, similar to effects observed with the antioxidant tiron (Figures 5(b) C, 5(b) D, and 5(c)). XCHT also dose-dependently reduced HG-stimulated ROS generation in the RMCs (Figure 5(c)). 

**Figure 6:**
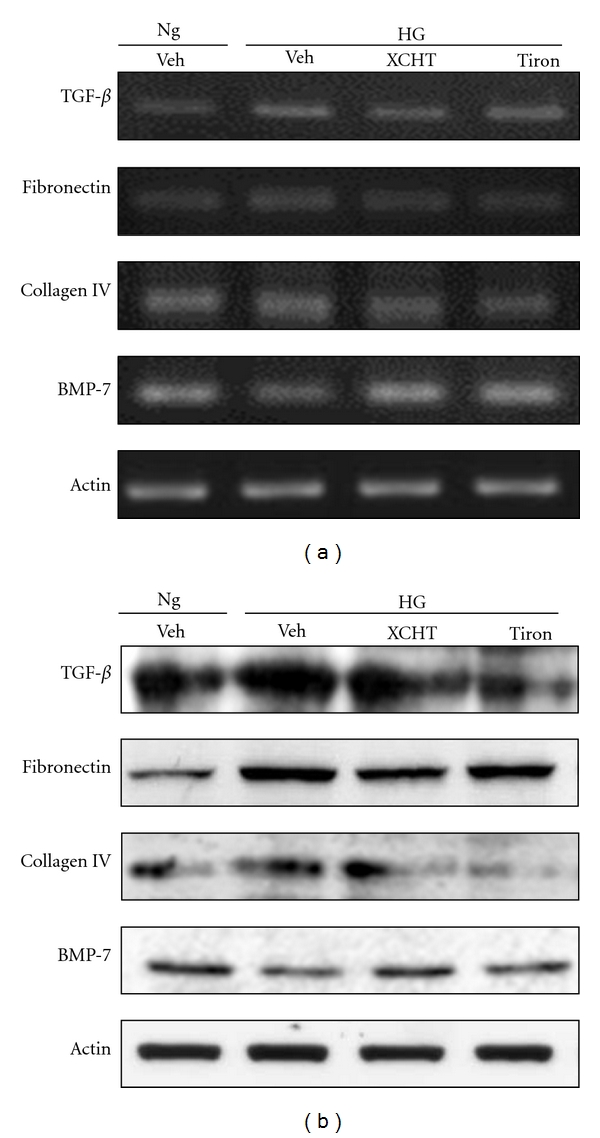
Effect of XCHT on TGF-*β*1, fibronectin, collagen IV, and BMP-7 expression in RMCs. RMCs were cultured in normal-glucose (NG; 5 mmol/L) or high-glucose (HG; 30 mmol/L) medium in the presence of 50 *μ*g/mL of XCHT for 24 h. (a) The mRNA expression of TGF-*β*1, fibronectin, collagen IV, and BMP-7 in RMCs detected by RT-PCR. *β*-Actin mRNA expression is used as an internal control. (b) Western blot analysis of TGF-*β*1, fibronectin, collagen IV, and BMP-7 expression in RMCs.

To assess whether the antioxidant effect from XCHT was related to its renal protective effects, we tested the mRNA and protein expression of TGF-*β*1, fibronectin, collagen IV, and BMP-7 in RMCs exposed to HG medium (Figures 6(a) and 6(b)). HG medium-treated RMCs showed lower levels of BMP-7 and increased TGF-*β*1, fibronectin, and collagen IV expression compared to NG medium-cultured RMCs. These effects, however, were reversed using the antioxidant tiron and XCHT treatments, whereby an enhancement in BMP-7 and a decrease TGF-*β*1, fibronectin, and collagen IV expressions were observed (Figures 6(a) and 6(b)). 

## 6. Discussion

Although the main clinical strategies for diabetic nephropathy (DN) are glycemic control and blood pressure regulation [3, 4], the therapeutic outcome remains unsatisfactory [5]. In the present study, we showed that XCHT could improve the renal functions and renal hypertrophy while decreasing serum glucose moderately in STZ-diabetic mice. Furthermore, we provide evidence that XCHT's renal protective mechanism in DN could potentially be mediated through (1) decreasing oxidative stress and production of TGF-*β*1, fibronectin, and collagen IV, and (2) increasing BMP-7 expression in the kidney of STZ-diabetic mice and HG-exposed RMCs. 

Recent search for new antifibrotic drugs has refocused on herbal medicine [27]. XCHT is widely prescribed to patients with liver cirrhosis for over a millennium in Asia, and the formula is established based on traditional clinical experience and practice [17, 18, 20]. TGF-*β*1 is involved in mediating the development of diabetic renal hypertrophy and is upregulated in diabetic kidney [28]. Hyperglycemia-induced upregulation of TGF-*β*1 stimulates mesangial cell proliferation and ECM induction (increased fibronectin and collagen production), which contributes to the major pathological changes observed in DN [28]. Thus, TGF-*β*1 has been considered as a therapeutic target in DN and other chronic kidney diseases [29]. XCHT has been documented to inhibit hepatic expression of TGF-*β*1 mRNA and protein in many experimental fibrogenic animal models [30, 31]. The *in vivo* results from the present study also indicated that XCHT treatment could decrease TGF-*β*1, fibronectin, and collagen IV expression in the kidney of STZ-diabetic mice. In addition, *in vitro*, the expression of these profibrotic proteins was also reduced by XCHT treatment in high-glucose-exposed RMCs. Thus, XCHT treatment appears to improve DN by reducing TGF-*β*1 and ECM production.

Oxidative stress induced by hyperglycemia is implicated in the development of DN [6]. ROS generated from high-glucose condition can stimulate a number of growth factors and cytokines, including TGF-*β*1, and lead to excessive production and accumulation of ECM proteins [6, 9, 32]. *In vitro* studies have shown that mesangial cells are the major source of free radicals after exposure to high-glucose concentrations [4, 33]. Previously, XCHT has also been reported to reduce the extracellular concentration of free radical by its O_2_^−^ and NO scavenging activity in LPS-stimulated cells [34]. Similarly, our results from the DPPH assay and intracellular ROS analyses have shown that not only XCHT could directly scavenge free radicals, but it could also decrease ROS generation in high glucose-exposed RMCs. The inhibition of ROS production by XCHT supposedly could prevent downstream stimulation of TGF-*β*1 activation and production, and ultimately avert the resulting aberrant ECM deposition. The *in vitro* results from the RMCs recapitulated our previous* in vivo* observations in the STZ-diabetic mice (Figures 3 and 4). Thus, it appears that the inhibitory effects of XCHT on high-glucose-induced ROS generation could potentially disturb stimulation of TGF-*β*1 and the resulting ECM proteins accumulation, thereby partly explaining the drug's renal protective effects in diabetic nephropathy. Thus, the observed effect of XCHT in decreasing the ECM proteins* in vitro *and* in vivo* is likely related to its antioxidative ability in impeding TGF-*β*1.

BMP-7 helps to maintain normal renal structure and physiological function, and its expression is decreased in damaged kidney [35]. During the development of DN, hyperglycemia causes oxidative stress and leads to BMP-7 expression decrease in renal cells [10–12]. Exogenous and overexpression of BMP-7 has been shown to improve renal function and prevent glomerular sclerosis in diabetic rats [12, 36]. The medicinal plant *Angelica sinensis* could also prevent STZ-diabetic rat from DN through increasing renal BMP-7 expression [37]. Our study showed that reduced renal BMP-7 expression in STZ-diabetic mice could be reversed by 4-week treatment with XCHT. Moreover, XCHT could increase BMP-7 expression in high-glucose-exposed RMCs. Our results therefore suggest that XCHT's protective effect against DN is at least in part mediated by increasing renal BMP-7 expression.

In conclusion, XCHT could prevent renal functions through augmenting BMP-7 expression and reducing ROS and ECM production. We suggest that XCHT could be applied for handling diabetic nephropathy and merits further study for its clinical application in diabetic nephropathy.

## Figures and Tables

**Table 1:** Constituents and proportions in 108 g of XCHT.

Plant name and part	Proportions (g)
*Bupleuri chinense* radix	28
*Glycyrrhizae* glabra L. radix	8
*Panax ginseng* C.A. Meyer radix	12
*Pinellia ternate* tuber	20
*Scutellaria baicalensis Georgi *radix	12
*Zingiberis officinale* Roscoe rhizoma	16
*Zizyphus vulgaris LAM *fructus	12
